# Modelled-Microgravity Reduces Virulence Factor Production in *Staphylococcus aureus* through Downregulation of *agr*-Dependent Quorum Sensing

**DOI:** 10.3390/ijms242115997

**Published:** 2023-11-06

**Authors:** Macauley J. Green, Ewan J. Murray, Paul Williams, Amir M. Ghaemmaghami, Jonathan W. Aylott, Philip M. Williams

**Affiliations:** 1 School of Pharmacy, University of Nottingham, University Park, Nottingham NG7 2RD, UK; macauley.green@nottingham.ac.uk (M.J.G.);; 2 Biodiscovery Institute and School of Life Sciences, University of Nottingham, University Park, Nottingham NG7 2RD, UKpaul.williams@nottingham.ac.uk (P.W.); 3 School of Life Sciences, University of Nottingham, University Park, Nottingham NG7 2RD, UK

**Keywords:** astropharmacy, *Staphylococcus aureus*, *agr*, quorum sensing, autoinducing peptide, microgravity, virulence, colonization, rotating cell culture system

## Abstract

Bacterial contamination during space missions is problematic for human health and damages filters and other vital support systems. *Staphylococcus aureus* is both a human commensal and an opportunistic pathogen that colonizes human tissues and causes acute and chronic infections. Virulence and colonization factors are positively and negatively regulated, respectively, by bacterial cell-to-cell communication (quorum sensing) via the *agr* (accessory gene regulator) system. When cultured under low-shear modelled microgravity conditions (LSMMG), *S. aureus* has been reported to maintain a colonization rather than a pathogenic phenotype. Here, we show that the modulation of *agr* expression via reduced production of autoinducing peptide (AIP) signal molecules was responsible for this behavior. In an LSMMG environment, the *S. aureus* strains JE2 (methicillin-resistant) and SH1000 (methicillin-sensitive) both exhibited reduced cytotoxicity towards the human leukemia monocytic cell line (THP-1) and increased fibronectin binding. Using *S. aureus agrP3::lux* reporter gene fusions and mass spectrometry to quantify the AIP concentrations, the activation of *agr*, which depends on the binding of AIP to the transcriptional regulator AgrC, was delayed in the strains with an intact autoinducible *agr* system. This was because AIP production was reduced under these growth conditions compared with the ground controls. Under LSMMG, *S. aureus agrP3::lux* reporter strains that cannot produce endogenous AIPs still responded to exogenous AIPs. Provision of exogenous AIPs to *S. aureus* USA300 during microgravity culture restored the cytotoxicity of culture supernatants for the THP-1 cells. These data suggest that microgravity does not affect AgrC-AIP interactions but more likely the generation of AIPs.

## 1. Introduction

Since early space exploration, it has been observed that many prokaryotes (bacteria) adapt and thrive in space vessels [1]. The space exposome subjects bacteria to conditions that are distinct from those encountered on Earth, such as increased radiation from cosmic rays, elevated carbon dioxide levels from human habitation of small, enclosed vessels and microgravity. As gravity has been a constant factor throughout the evolution of every organism on Earth, exposure to the near-absence of gravity constitutes a novel environmental experience for an organism.

Since it is not feasible to completely sterilize an entire space vessel or to maintain a sterile environment for manned spaceflights given the human microbiome, NASA (National Aeronautics and Space Administration) has set microbial acceptability limits for space missions [1]. Most bacteria have short growth cycles and readily adapt to environmental changes. As space is a novel environment for bacteria, it is difficult to predict how they will adapt and whether a particular species might pose an increased threat to human health. This need to understand bacterial adaptation becomes especially important as the duration of missions extend, such as longer term habitation of the International Space Station (ISS), extended habitation of the lunar surface and extra-terrestrial space station (Lunar Gateway) and possible manned missions to Mars.

In over 60 years of space exploration there have been several missions in which bacterial adaptive behavior has been investigated [1]. By combining ground-based analogue with spaceflight data it is possible to investigate how different bacterial species adapt to microgravity, and to begin to understand the mechanisms behind these adaptative processes. However, a major factor in studying bacterial adaptation to microgravity is that there is no universal bacterial response to microgravity [2] although most microorganisms thrive in microgravity during spaceflight [3].

Bacterial contamination can be reduced for space missions by countermeasures such as pre-flight quarantine of astronauts and freeze-drying food [4]. To meet NASA pre-flight microbial acceptability limits, viable counts are conducted on random samples of air, water and surfaces [1]. Data from over 100 missions have been recorded and the diversity of microbial contaminants found upon/in spacecraft determined [5]. The clean rooms of European Space Agency (ESA) member states in which space vessels are assembled were found to contain 298 bacterial strains presenting a diverse population of potential contaminants [6].

The most common bacterial species encountered in the air and on the surfaces of manned space vessels are the staphylococci [7]. *Staphylococcus aureus* is a Gram-positive bacterium that is commonly found in the environment (soil, water and air) and can cause both minor and severe infections [8]. It is a member of the normal human flora, mainly residing on skin and mucous membranes such as those of the nasal cavity [8]. *S. aureus* colonizes around 30% of the human population [9], is a leading cause of bacteremia (viable bacteria in the blood [10]) and is also responsible for lung, bone, joint, skin, soft tissue and surgical site infections [11]. Treatment of *S. aureus* infections has been complicated by the emergence of vancomycin (VRSA)- and methicillin (MRSA)-resistant strains. MRSA strains can be sub-divided into hospital-acquired (HA-MRSA) and community-acquired (CA-MRSA) [12]. While HA-MRSA strains are generally of low virulence and cause infections in hospitalized or immune-compromised individuals, CA-MRSA strains combine multi-antibiotic resistance with high virulence and transmissibility, enabling them to spread and cause severe infections in healthy individuals [13]. *S. aureus* is therefore likely to pose a significant infection risk for astronauts during manned space travel. It is currently on the World Health Organization (WHO) list of high priority, antibiotic resistant pathogens for which new antimicrobial therapies are urgently required [12].

While colonization generally refers simply to the presence of bacteria on a body surface, infection occurs when bacteria penetrate host tissues and engage with host immune defences [14]. *S. aureus* produces diverse cell wall-associated host colonization factors and immune modulating agents and secretes multiple virulence determinants that include tissue-damaging exotoxins and exoenzymes [13]. Colonization factors include immunoglobulin-, fibrinogen- and fibronectin-binding proteins that facilitate attachment to host cell surfaces and block phagocytosis [13]. Secreted virulence determinants include cytotoxins such as α-hemolysin which cause extensive host cell lysis and tissue damage while others (e.g., toxic shock syndrome toxin) function as super-antigens promoting the onset of shock-like syndromes [13].

In many bacterial pathogens including *S. aureus*, virulence factor production is controlled by quorum sensing (QS). This is a regulatory mechanism that enables unicellular micro-organisms to synchronize gene expression within a population through cell-to-cell communication via the production and sensing of a self-generated, diffusible, signal molecule [15]. As the number of bacteria in a growing population increases, the extracellular concentration of the QS signal molecule will rise. Once a threshold concentration (quorum) has been reached, a sensor kinase or response regulator protein is activated (or repressed), so controlling the expression of QS-dependent genes [15]. The size of the quorum is not fixed but depends on the relative rates of QS signal molecule production, diffusion and turnover [15]. QS signal molecules are often called ‘autoinducers’ as they are responsible for driving their own biosynthesis [15].

**Figure 1:**
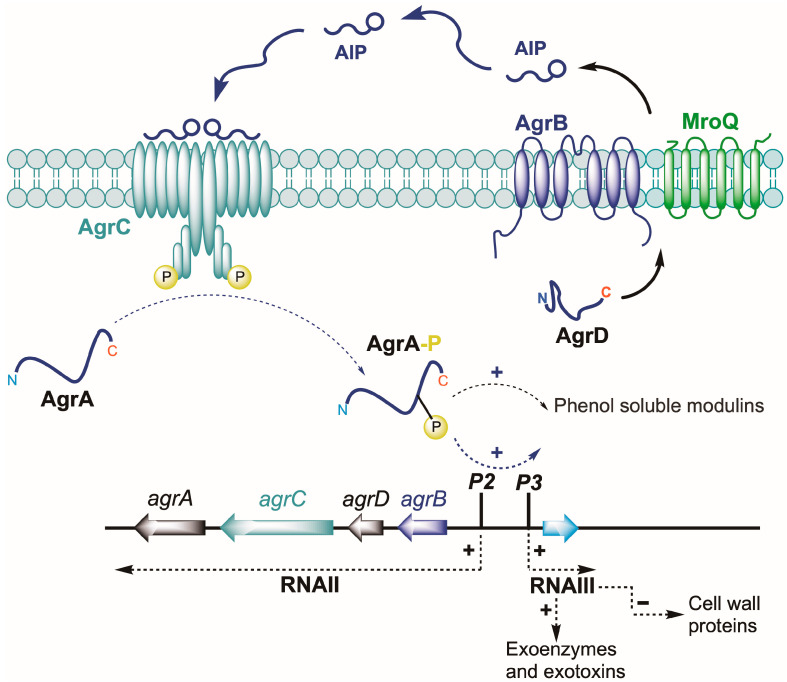
The *agr* QS locus in *S. aureus* consists of two divergent transcripts consisting of *agrBDCA* (RNAII) and the regulatory RNA, RNAIII, that also codes for δ-hemolysin. The *agrB* gene codes for a transmembrane peptidase, that together with a second transmembrane peptidase (MroQ) processes AgrD to generate the autoinducing peptide (AIP). Once released extracellularly, the AIP binds to the histidine sensor kinase, AgrC, on neighboring cells resulting in phosphorylation and subsequent phosphotransfer to AgrA. This leads to activation of the *agr*P2 and *agr*P3 promoters driving the autoinduction circuitry to generate more AIPs so inducing the QS-dependent up-regulation of many exoproduct virulence genes and the down-regulation of cell wall colonization factors. (reproduced from [17]).

In Gram-positive bacterial pathogens including the staphylococci, clostridia, enterococci and listeria, QS is mediated by the accessory gene regulator (*agr*) system [16,17] which reciprocally controls colonization and virulence gene expression. In *S. aureus*, the *agr* locus consists of two divergent transcriptional units (*agrBCDA* and RNAIII), controlled by the *agr*P2 and *agr*P3 promoters, respectively [17], (Figure 1).

AgrC and AgrA function as sensor kinase and response regulator proteins, respectively, while the QS signal molecule, an autoinducing peptide (AIP), is generated via the AgrB- and MroQ-dependent proteolytic processing of the AgrD pro-peptide. The binding of an autoinducing peptide (AIP) to AgrC leads to the phosphorylation of AgrA, which in turn activates the *agr*P2 promoter. This upregulates *agrBCDA* expression conferring a positive feedback loop that autoinduces AIP production via AgrB and AgrD and activates the *agr*P3 promoter driving downstream virulence gene expression primarily via RNAIII [17,18].

Early studies of *Salmonella typhimurium* grown on board a space shuttle revealed global changes in bacterial gene expression and increased virulence in a mouse infection model compared with ground controls [19]. A similar increase in virulence was also observed by growing *Salmonella* in NASA-designed rotating cell culture system (RCCS) bio-reactors [19]. These maintain bacterial cells in suspension while exposing them to a low-shear modelled microgravity environment (LSSMG) [19]. Compared with the controls, *S. aureus* strains grown under LSMMG have been reported to produce reduced levels of hemolysins, carotenoid pigments, an enhanced susceptibility to oxidative stress, increased susceptibility to killing by whole human blood and differential regulation of numerous metabolic genes [20,21] However, the molecular mechanism(s) responsible for these diverse changes has not been elucidated. Given that the *S. aureus agr* system regulates colonization and virulence factor production and mediates protection from lethal oxidative stress [17,22], we sought to determine whether *agr*-dependent QS is modulated by growth under LSMMG culture conditions. For both MRSA and methicillin-sensitive *S. aureus* (MSSA) strains, *agr* expression was delayed and AIP production reduced under LSMMG via a mechanism that involves AIP generation rather than AIP-dependent activation of AgrC.

## 2. Results

**Figure 2:**
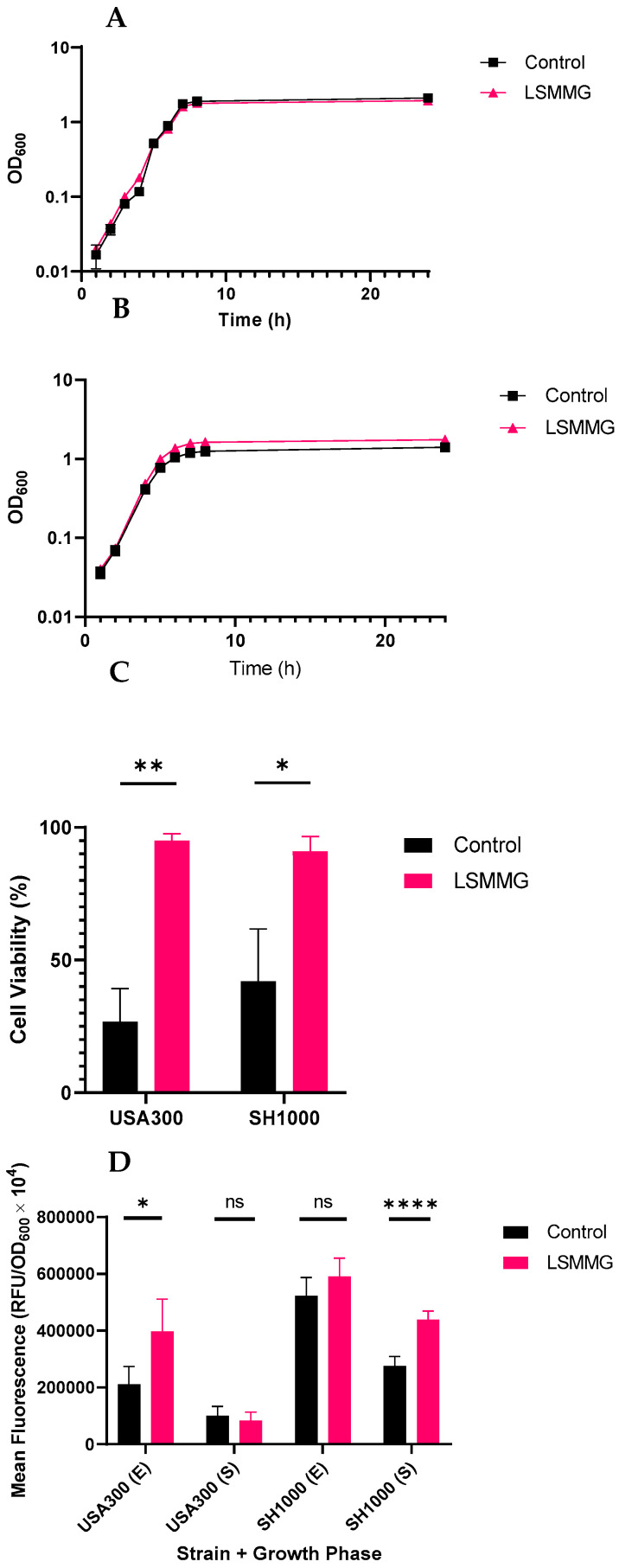
Growth, cytotoxicity and fibronectin-binding by *S. aureus* JE2 and SH1000 strains grown in RCCS vessels under normal (control) and LSMMG conditions. (**A**,**B**) growth curves, (**C**) cytotoxicity of cell-free culture supernatants, bars show THP-1 cell viability after incubation with bacterial cell-free culture supernatants and (**D**) binding of *S. aureus* cells to fibronectin-coated microtiter plate wells. * *p* < 0.05; ** *p* < 0.005; **** *p* < 0.00005, ns, not significant. Exponential phase samples were taken after 4 h and stationary phase samples after 24 h of growth. Independent *t*-tests were performed using GraphPad. All experiments were carried out in triplicate. Error bars show standard deviation of the mean.

To investigate the impact of LSMMG on *agr*-dependent QS in *S. aureus*, we selected the highly virulent community-acquired USA300 MRSA strain, JE2 (Table 1), and the well-studied MSSA laboratory strain, SH1000 (Table 1), both of which produce AIP-1. Figure 2 shows their growth (Figure 2A,B), cytotoxicity for the human leukemia monocytic cell line (THP-1) (Figure 2C) and attachment to a fibronectin-coated surface (Figure 2D) under both control and LSMMG conditions. We observed that the LSMMG culture did not affect the growth (Figure 2A,B) or cell morphologies of either strain (Figure S1).

Exotoxin production and hence cytotoxicity for mammalian cells is up-regulated by the *agr* system which down-hand colonization [17]. To quantify the overall impact of LSMMG growth conditions on the production of lytic *S. aureus* exotoxins, we used a well-established assay employing the immortalized T-cell line, THP-1 [28,29]. Incubation of cell-free *S. aureus* culture supernatants with THP-1 cells followed by determination of THP-1 cell survival provides a quantitative assay for multiple *agr*-dependent exotoxins including β-hemolysin, δ-hemolysin and γ-hemolysin as well the leukocidins and phenol-soluble modulins [28,29]. Figure 2C shows that following growth under control conditions, culture supernatants prepared from both JE2 and SH1000 were highly cytotoxic for the THP-1 cells. However, the supernatants from both strains cultured under LSMMG conditions exhibited negligible cytotoxicity.

Conversely, the binding of exponential *S. aureus* JE2 cells to fibronectin, which depends primarily on the cell wall fibronectin-binding proteins, FnbA and FnbB [30] increased after growth under LSMMG conditions compared with the control conditions (Figure 2D). For the exponential SH1000 cells, fibronectin binding was higher than for the exponential JE2 cells but there was little difference between the LSMMG and control cultures. A significant difference was however noted for the stationary phase SH1000 but not for the JE2 (Figure 2D).

**Figure 3:**
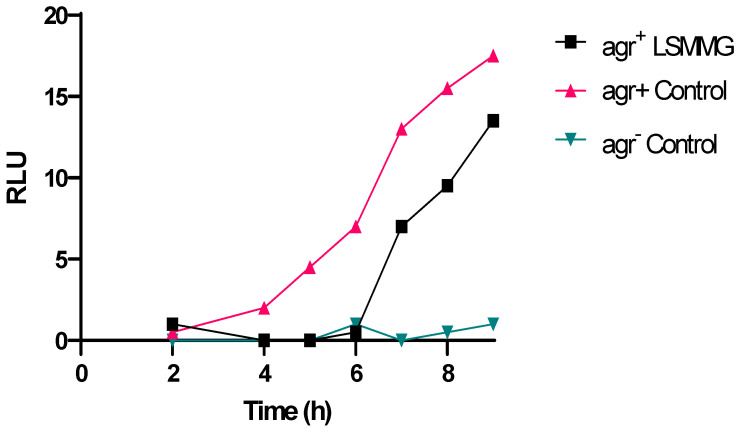
Activation of *agr*P3*::lux* during growth of *S. aureus* JE2 under control and LSMMG conditions. The bioluminescence of USA300 *agrP3::lux* is shown over time in X-Vivo 15 medium and quantified as Relative Light Units (RLU). An *agr* deletion mutant, *S. aureus* JE2 Δ*agr agr*P3::*lux* was used as a negative control for background bioluminescence. Experiments were carried out in triplicate.

Given that the results presented in Figure 2C,D are consistent with reduced expression of the *agr* system when *S. aureus* was grown under the LSMMG conditions compared with the controls, we examined the timing of *agr* activation as a function of growth and quantified the AIP production. By integrating an *agrP3::lux* transcriptional reporter fusion into an intergenic *attB2* site on the chromosome of *S. aureus*, the expression of *agr* can be followed in real time [26] (Table 1). Figure 3 shows that when *S. aureus* JE2 *attB agrP3*::*lux* was grown in X-VIVO medium under LSMMG, *agr* expression was delayed by ~4 h compared with the control. This difference in the timing of *agr*P3 expression was highly likely to have contributed to the reduced exotoxin and increased fibronectin-binding observed in Figure 2C,D.

**Figure 4:**
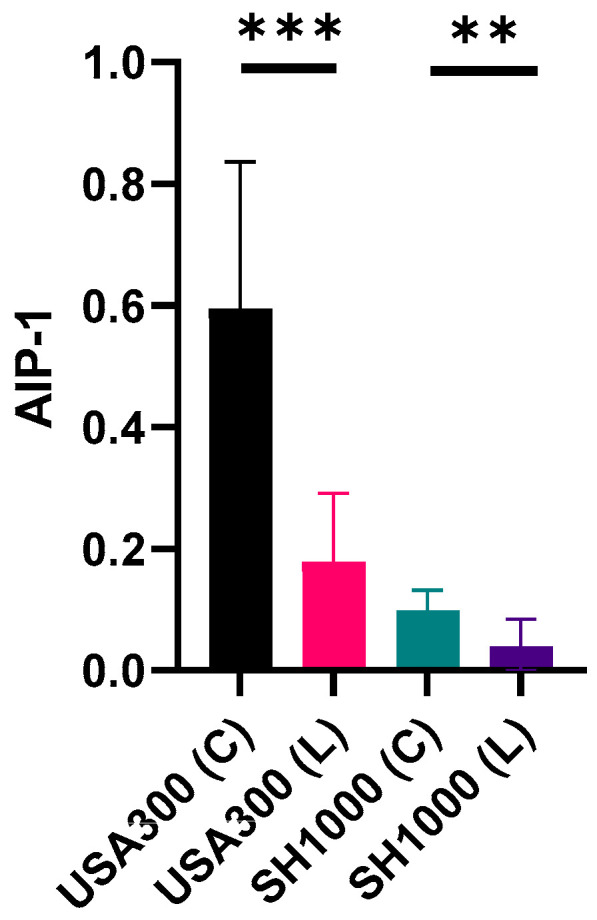
AIP production by *S. aureus* strains JE2 and SH1000 grown in X-Vivo 15 medium to stationary phase in RCCS under both control (C) and LSMMG (L) conditions. AIP concentrations (µM) were quantified using LC MS/MS. Independent *t*-tests were performed using GraphPad. Experiments were carried out in triplicate. Error bars show standard deviation of the mean. ** *p* < 0.005; *** *p* < 0.0005.

To determine whether the differential expression of *agr* observed in Figure 3 resulted in reduced AIP-1 accumulation, the AIP concentrations in cell-free, stationary phase culture supernatants were quantified by liquid chromatography tandem mass spectrometry (LC MS-MS). Figure 4 shows that *S. aureus* JE2 produced ~30% less AIP-1 in LSMMG culture (0.18 µM) compared with the control culture (0.6 µM). For *S. aureus* SH1000, an AIP-1 reduction of ~40% was observed (Figure 3).

**Figure 5:**
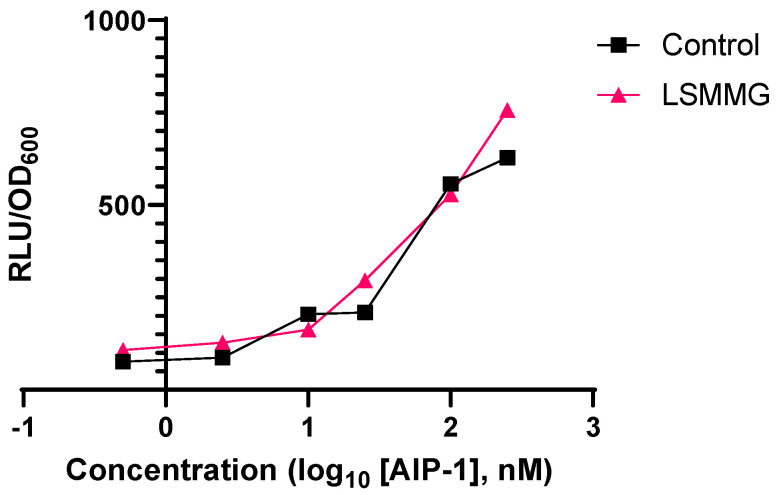
Dose–response curves for the activation of *lux*-based *agr*P3 reporter strain ROJ143 by exogenous synthetic AIP-1 under both control and LSMMG growth. Experiments were carried out in triplicate.

The *agr* system is autoinducible and depends on an *S. aureus* culture reaching a threshold concentration of AIP that facilitates the population-wide activation of AgrC [17]. The observed delay in *agr* expression and reduction in AIP-1 production when *S aureus* was grown under LSMMG conditions may therefore relate to a change in the response of AgrC1 to AIP-1. To explore this possibility, we used the *S. aureus agrP3*::*lux* reporter strain ROJ143 in which the AIP biosynthesis genes *agrB* and *agrD* have been deleted [26,27] (Table 1). ROJ143 cannot produce AIP-1 but is sensitive to exogenously supplied AIP-1 such that the AgrC/AIP interaction activates *agrP3::lux* resulting in the production of light. When ROJ143 was grown in the presence of a range of concentrations of exogenous synthetic AIP-1 no differences in light output were observed indicating that growth under LSMMG did not impact on AIP-dependent AgrC activation (Figure 5).

**Figure 6:**
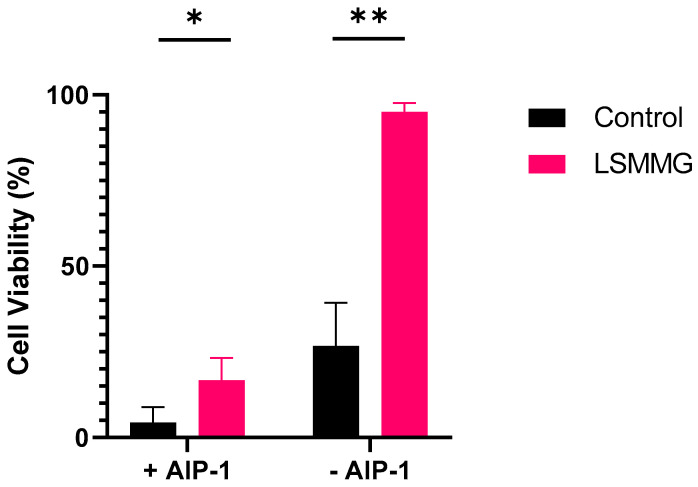
Viability of THP-1 cells exposed to stationary phase cell-free supernatants from *S. aureus* JE2 grown with (+) or without (−) AIP-1 (100 nM). Independent *t*-tests were performed using GraphPad. Experiments were carried out in triplicate. Error bars show standard deviation of the mean. * *p* < 0.05; ** *p* < 0.005.

To determine whether exogenous AIP could restore the cytotoxicity of JE2 during growth under LSMMG conditions, we grew JE2 in the RCCS in the presence of AIP-1. Figure 6 shows that provision of AIP-1 at the time of inoculation reduced THP-1 cell viability from 90% to 15%. Under control growth conditions, exogenous AIP-1 reduced THP-1 viability from 25% to 4% (Figure 6). These data confirm that the lack of AIP rather than the inability to respond to AIP appears to be responsible for the reduced cytotoxicity observed under LSMMG conditions.

## 3. Discussion

When cultured under LSMMG conditions, bacterial pathogens respond differentially when compared with controls [31]. Changes in growth dynamics, gene expression, cellular aggregation, attachment, biofilm formation, conjugation, antimicrobial susceptibility and virulence (either enhanced or reduced) have all been observed [31] The impact of microgravity on the virulence of several Gram-positive bacterial pathogens has been investigated by utilizing nematode killing assays [32]. In these, spaceflight was associated with reduced killing of both larval and adult *Caenorhabditis elegans* nematodes by *S. aureus*, *Listeria* and *Enterococcus* strains. For *S. aureus*, the reported behavior of different MSSA and MRSA strains when cultured in rich laboratory media under LSMMG conditions compared with controls was broadly similar with respect to growth kinetics; either minor differences or none were noted in agreement with the present study [21,33]. Further investigation of an HA-MRSA strain, N315, showed that when grown under LSMMG, N315 responded by adopting a colonization/biofilm phenotype as reflected by the formation of cell aggregates encased in an extracellular polymeric substance, reduced production of the yellow carotenoid pigment staphyloxanthin and exhibition of a four-fold increase in susceptibility to hydrogen peroxide during the early stages of LSMMG culture [20]. This increased susceptibility may be linked in part to a reduction in staphyloxanthin production which neutralizes reactive oxygen species such as those produced by neutrophils [34]; data that is consistent with the reduced survival of N315 in whole human blood [20]. Similar results for staphyloxanthin production were observed for three MSSA clinical isolates by Rosado et al. [21], who also observed reduced hemolysin production under LSMMG conditions compared with controls. Micro-array-based transcriptome analysis of MSSA strain RF6 [21] confirmed the reduced expression of the *hla* gene that codes for α-hemolysin as well as statistically significant reductions in regulatory genes including *saeRS* and *sarA* which, in addition to *agr*, are involved in the control of *hla* expression [35,36]. For HA-MRSA strain N315 grown in LSMMG, the only significantly downregulated global regulatory element was *hfq* [20]. This gene codes for an RNA-binding protein that stabilizes mRNA transcripts, so facilitating post-translational regulation. Hfq has been associated with differential virulence or virulence gene expression in both space flight and LSMMG growth conditions for the Gram-negative pathogens *S. typhimurium* [19] and *Pseudomonas aeruginosa* [37], although the regulatory pathway(s) linking the sensing of microgravity to the activity of Hfq has not been identified [31]. Although Hfq has a well-defined global regulatory role in *S. typhimurium* and *P. aeruginosa*, its contribution to post-transcriptional gene regulation and the virulence of *S. aureus* has not been unequivocally demonstrated [38].

In *S. aureus*, the *agr* QS system controls the expression of over 200 genes and is characterized by the increased production of tissue-damaging exotoxins and exoenzymes at the onset of the stationary phase while reducing the expression of genes coding for cell wall proteins involved in host cell attachment and evasion of host defenses [17,35,39,40,41,42,43]. The *S. aureus* transcriptome analysis carried out by Castro et al. [20] did not reveal any differential regulation of the *agr* system nor of any *agr* target genes following growth in LSMMG. However, Rosado et al. [21] provided evidence for downregulation of *hla* and *sarA*, the product of which controls expression of the *agr* system via the *agr*P2 promoter [35]. Our data showing the downregulation of exotoxins and increased production of fibronectin binding after growth under LSMMG conditions is therefore consistent with the findings of Rosado et al. [21] at least for the CA-MRSA and MSSA strains evaluated. The *S. aureus* strain N315 used by Castro et al. [20] is an HA-MRSA strain which in common with many HA-MRSA strains is only weakly cytotoxic [44,45]. For certain HA-MRSA strains, reduced *agr* activation has been linked to changes in the cell wall architecture associated with acquisition of the mobile genetic element (SCC*mec*) that confers methicillin resistance [45].

Since the LSMMG environment is low-shear, the reduced external gravitational forces on the bacterial cell may not counter-balance the internal turgor pressure such that staphylococcal growth and/or cell morphology may be affected. Neither the CA-MRSA strain JE2 nor the MSSA strain SH1000 exhibited any changes that would explain the effect of LSMMG on fibronectin-binding or cytotoxicity that we observed. However, analysis of *agr* expression as a function of growth together with quantification of AIP-1 revealed that LSMMG affected the timing of *agr* expression and reduced the accumulation of AIP-1. The induction of *agr* in JE2 was delayed by ~4 h until the cells were approaching the end of the exponential phase (between 6 and 8 h). Although the AIP-1 concentration may have reached the threshold required for induction of the *agr* QS system in *S. aureus* JE2, it is important to note that *agr* is subject to complex control by numerous other regulators, repressors and alternative sigma factors, including the Sar proteins, Rot and MgrA [35,39] as well as via regulatory systems that sense perturbations in staphylococcal metabolism [46]. Consequently, there is a ‘window period’ of responsiveness during the exponential phase of growth after which AIP concentrations at or above the threshold fail to activate the *agr* circuitry [26]. This is clearly apparent in certain *S. aureus* clinical isolates which carry mutations in the C-terminal domain of the AIP-1 receptor, AgrC1 [26].

This raises the question of why LSMMG culture resulted in such a significant delay in the activation of *agr*. To address this question, we conducted experiments to determine whether LSMMG culture influenced the dose-dependent activation of the AgrC1 receptor by AIP-1. To uncouple the AIP-dependent *agr* autoinduction circuitry and to facilitate investigation of the AgrC1 response in the absence of AIP-1 biosynthesis, we used the *S. aureus* strain ROJ143 in which *agrD* and *agrB* have been deleted. This strain carries a chromosomally located *agr*P3::*lux* reporter fusion and is dark in the absence of AIP-1 but emits light in response to exogenously supplied AIP-1. ROJ143 is highly responsive to AIP-1 with an EC_50_ of 3.2 nM [27]. When grown under LSMMG conditions and compared with the controls, there were no differences in the response of ROJ143 to a range of concentrations of AIP-1 from 0.5 to 250 nM. These data suggested that the responsiveness of AgrC1 to AIP-1 was not affected by LSMMG. Furthermore, the exogenous provision of AIP-1 at the point of inoculation fully restored the cytotoxicity for THP-1 cells of supernatants prepared from both JE2 and SH1000 cultured under LSMMG. Exogenous AIP-1 is therefore capable of activating both *agr* and its downstream hemolysin target genes under LSMMG conditions.

AgrC–AIP interactions are therefore very unlikely to be responsible for the impact of LSMMG on *agr*-dependent QS. A plausible alternative involves reduced biosynthesis, increased turnover or reduced export of AIP-1. We have previously shown that AIP-1 is readily oxidized in batch cultures forming an inactive sulphoxide [47] However, given that the RCCS is a closed system, it is unlikely that oxygenation under LSMMG and control conditions is different. AIP biosynthesis depends on the proteolytic processing of the AgrD pro-peptide by the transmembrane proteases AgrB and MroQ via at least four membrane-associated stages. These stages are as follows: (i) removal of a C-terminal peptide from AgrD, (ii) formation of the thiolactone macrocycle, (iii) cleavage of an N-terminal peptide AgrD and (iv) export of the AIP [17,18,48]. Given that AgrD processing is membrane-associated and involves transmembrane protein–protein interactions, it is possible that LSMMG influences AIP generation through changes in membrane fluidity or stability as have been noted for eukaryotic cells [49] and other bacteria [50], respectively. Further work will be required to unravel the molecular mechanism by which LSMMG downregulates *agr* expression with a focus on AIP biosynthesis and whether this occurs during spaceflight.

## 4. Materials and Methods

### 4.1. Bacterial Strains and Growth Media

The *S. aureus* strains used in this study are listed in Table 1. Bacteria were routinely grown in tryptic soy broth (TSB) at 37 °C for 16 h with shaking at 200 rpm or on tryptic soy agar (TSA) at 37 °C for 24 h. *S. aureus* was also cultured in X-Vivo 15 serum-free medium without gentamicin or phenol red (Lonza, Slough, UK). For the RCCS experiments, *S. aureus* was cultured in 2 mL autoclavable vessels (Synthecon, Houston, TX, USA) incubated at 37 °C in a 5% CO_2_ atmosphere. Samples were rotated at 25 rpm in a vertical orientation for LSMMG cultures and horizontal orientation for control cultures. Bacterial growth was recorded by taking a 100 µL sample every h for the first 8 h and at 24 h. This was added to 900 µL X-Vivo15 medium in a quartz cuvette and optical density at 600 nm (OD_600_) determined.

### 4.2. Cell Attachment

Bacterial cell attachment assays were performed by adding 1 × 10^6^ cells from either 4 h or 24 h growth of *S. aureus* JE2 and SH1000 tagged with *gfp* (Table 1) to wells in a fibronectin coated 96-well plate. Plates were incubated at 37 °C for 30 min and washed three times with phosphate buffered saline (PBS) OD_600_ and fluorescence at 488 nm was recorded in a microplate reader (TECAN, Männedorf, Switzerland).

### 4.3. THP-1 Cytotoxicity

Cytotoxicity was determined by measuring the viability of THP-1 cells (human leukemia monocytic cell line) exposed to cell-free *S. aureus* culture supernatants as described in Collins et al. [28] and Labeei et al. [29]. THP-1 cells were cultured in RPMI-1640 medium overnight and 1 × 10^6^ cells removed and added to each well in a 24-well plate. Cell-free culture supernatants from JE2 or SH1000 cultures were added to the THP-1 cells and cell viability was measured by adding trypan blue in a 1:1 ratio, adding 10 µL of this solution onto a hemocytometer slide and recording percentage viability on an automated cell counter (Countess, ThermoFisher, Waltham, MA, USA).

### 4.4. Activation of agr in S. aureus JE2

The activation of *agr* over time was quantified by sampling 100 µL of *S. aureus* JE2 *agr*P3::*lux* cultures every hour between 2 h and 9 h of growth. An amount of 100 µL of fresh medium was added to the culture vessels for each sample taken. *S. aureus* JE2 *agr*P3::*lux* was cultured in a 2 mL HARV vessel on an RCCS in either a horizontal or vertical orientation. The medium used for this was X-Vivo 15 serum-free medium (Lonza). Samples were pipetted into a 96-well plate luminescence and OD_600_ were recorded using a microplate reader (TECAN).

### 4.5. AIP Detection and Quantification Using ROJ143

Cultures containing AIPs were centrifuged after 24 h and the cell-free supernatant collected. For the *agr* bioreporter assay [51], 5 µL of supernatant was added to a 1:250 dilution of overnight ROJ143 cultured in a 96-well plate in triplicate. This was placed in a microplate reader (TECAN) for 16 h at 37 °C and luminescence and OD_600_ recorded every 15 min. When required for quantification, the area under the curve for each timepoint was calculated and plotted using GraphPad.

### 4.6. Mass Spectrometry

Chromatography was achieved using a Shimadzu series 10AD VP LC system. The column oven was maintained at 40 °C. The HPLC Column used was a Kinetex core-shell XB-C18 (2.6 µm; 50 mm × 3.0 mm) with an appropriate guard column. Mobile phase A was 0.1% (*v*/*v*) formic acid in water, and mobile phase B 0.1% (*v*/*v*) formic acid in methanol. The flow rate throughout the chromatographic separation was 450 µL/min. The binary LC gradient initially began at 10% B for 1.0 min, and increased linearly to 99% B over 5.0 min. The composition remained at 99% B for 1.0 min, decreased to 10% B over 0.5 min, and stayed at this composition for 3.5 min. The total run time per sample was 10 min.

The MS system used was an Applied Biosystems Qtrap 4000 hybrid triple-quadrupole linear ion trap mass spectrometer equipped with an electrospray ionization (ESI) interface. Instrument control, data collection and analysis were conducted using Analyst software (v1.6.3). Source parameters were set as: curtain gas: 25.0, ion source potential: 5000 V, temperature: 450 °C, nebulizer gas: 20.0, auxiliary gas: 20.0, and entrance potential: 10 V. The MS detection was conducted with the MS operating in MRM (multiple reaction monitoring) mode. Positive electrospray conditions (+ES) conditions were used based upon the parameters published by Junio et al. [52], using a [M + H]^+^ precursor ion of *m*/*z* = 961 with a product ion of *m*/*z* = 711 following collision induced dissociation (CID). An amount of 10 µL of the sterile filtered cell-free bacterial supernatant samples, without any further sample preparation, were analyzed using the developed LC-MS/MS methodology. Biological samples were run in a single batch of samples alongside calibration samples of a synthetic standard of AIP-1 (synthesized described by McDowell et al. [47] prepared in MeOH, ranging from 0 nM to 1000 nM. 

### 4.7. Activation of agr via Exogenous AIP-1

For exogenous AIP studies, synthetic AIP-1 was added at a range of concentrations (0.5 nM–250 nM) to ROJ143 immediately prior to inoculation of *S. aureus* in X-Vivo 15 medium (Lonza). Bacteria were cultured in an RCCS in either a horizontal or vertical orientation. ROJ143 is an engineered *S. aureus* strain that emits light when the *agr* operon is activated by AIP-1 but cannot produce endogenous AIPs [27]. Samples of 100 µL were taken every hour between 2 and 8 h and pipetted into a 96-well plate in a TECAN plate reader where luminescence and OD_600_ was recorded. For every sample taken, 100 µL of fresh medium containing the same concentration of AIP-1 was added.

## Figures and Tables

**Table 1:** Bacterial strains used in this study.

Strain	Description	Reference
JE2	Plasmid-cured derivative of the CA-MRSA USA300 LAC; *agr* group I strain	[23,24]
SH1000	MSSA strain; functional *rsbU* derivative of 8325-4, *rsbU*^+^; *agr* group I strain	[25]
ROJ143	RN4220 *agr::tetM agr*P3*:luxCDABE pSKermP2:agrC1 agrA*	[26,27]
USA300 *gfp*	pSB2019 (P*_xylA_::gfp*)	This laboratory
SH1000 *gfp*	pSB2019 (P*_xylA_::gfp*)	This laboratory
USA300 *agrP3*::*lux*	Luminescent strain of USA300 with *attB2: agrP3::luxCDABE*	This laboratory
USA300 Δagr *agr*P3::lux	*agr* deletion mutant with *attB2: agrP3::luxCDABE*	This laboratory

## Data Availability

Research data is stored at the University of Nottingham and can be made available upon request.

## Supplementary Materials

The following supporting information can be downloaded at: https://www.mdpi.com/article/10.3390/ijms242115997/s1.

## References

[1] Green M.J., Aylott J.W., Williams P., Ghaemmaghami A.M., Williams P.M. (2021). Immunity in space: Prokaryote adaptations and immune response in microgravity. Life.

[2] Sharma G., Curtis P.D. (2022). The impacts of microgravity on bacterial metabolism. Life.

[3] Horneck G., Klaus D.M., Mancinelli R.L. (2010). Space microbiology. Microbiol. Mol. Biol. Rev..

[4] Bijlani S., Stephens E., Singh N.K., Venkateswaran K., Wang C.C.C. (2021). Advances in space microbiology. iScience.

[5] Pierson D.L. (2001). Microbial contamination of spacecraft. Gravit. Space Biol. Bull..

[6] Moissl-Eichinger C., Rettberg P., Pukall R. (2012). The first collection of spacecraft-associated microorganisms: A public source for extremotolerant microorganisms from spacecraft assembly clean rooms. Astrobiology.

[7] Yamaguchi N., Roberts M., Castro S., Oubre C., Makimura K., Leys N., Grohmann E., Sugita T., Ichijo T., Nasu M. (2014). Microbial monitoring of crewed habitats in space-current status and future perspectives. Microbes Environ..

[8] Taylor T.A., Unakal C.G. (2022). Staphylococcus aureus.

[9] Wertheim H.F., Melles D.C., Vos M.C., van Leeuwen W., van Belkum A., Verbrugh H.A., Nouwen J.L. (2005). The role of nasal carriage in *Staphylococcus aureus* infections. Lancet Infect. Dis..

[10] Smith D.A., Nehring S.M. (2022). Bacteremia.

[11] Tong S.Y., Davis J.S., Eichenberger E., Holland T.L., Fowler V.G. (2015). *Staphylococcus aureus* infections: Epidemiology, pathophysiology, clinical manifestations, and management. Clin. Microbiol. Rev..

[12] Chambers H.F., Deleo F.R. (2009). Waves of resistance: *Staphylococcus aureus* in the antibiotic era. Nat. Rev. Microbiol..

[13] Cheung G.Y.C., Bae J.S., Otto M. (2021). Pathogenicity and virulence of *Staphylococcus aureus*. Virulence.

[14] Howden B.P., Giulieri S.G., Wong Fok Lung T., Baines S.L., Sharkey L.K., Lee J.Y.H., Hachani A., Monk I.R., Stinear T.P. (2023). *Staphylococcus aureus* host interactions and adaptation. Nat. Rev. Microbiol..

[15] Williams P., Winzer K., Chan W.C., Camara M. (2007). Look who’s talking: Communication and quorum sensing in the bacterial world. Philos. Trans. R. Soc. Lond. B Biol. Sci..

[16] Gray B., Hall P., Gresham H. (2013). Targeting *agr*- and *agr*-like quorum sensing systems for development of common therapeutics to treat multiple gram-positive bacterial infections. Sensors.

[17] Williams P., Hill P., Bonev B., Chan W.C. (2023). Quorum-sensing, intra- and inter-species competition in the staphylococci. Microbiology.

[18] Bardelang P., Murray E.J., Blower I., Zandomeneghi S., Goode A., Hussain R., Kumari D., Siligardi G., Inoue K., Luckett J. (2023). Conformational analysis and interaction of the *Staphylococcus aureus* transmembrane peptidase AgrB with its AgrD propeptide substrate. Front. Chem..

[19] Wilson J.W., Ott C.M., Honer zu Bentrup K., Ramamurthy R., Quick L., Porwollik S., Cheng P., McClelland M., Tsaprailis G., Radabaugh T. (2007). Space flight alters bacterial gene expression and virulence and reveals a role for global regulator Hfq. Proc. Natl. Acad. Sci. USA.

[20] Castro S.L., Nelman-Gonzalez M., Nickerson C.A., Ott C.M. (2011). Induction of attachment-independent biofilm formation and repression of Hfq expression by low-fluid-shear culture of *Staphylococcus aureus*. Appl. Environ. Microbiol..

[21] Rosado H., Doyle M., Hinds J., Taylor P. (2010). Low-shear modelled microgravity alters expression of virulence determinants of *Staphylococcus aureus*. Acta Astronaut..

[22] Podkowik M., Perault A.I., Putzel G., Pountain A., Kim J., Dumont A., Zwack E., Ulrich R.J., Karagounis T.K., Zhou C. (2023). Quorum-sensing agr system of *Staphylococcus aureus* primes gene expression for protection from lethal oxidative stress. eLife.

[23] McDougal L.K., Steward C.D., Killgore G.E., Chaitram J.M., McAllister S.K., Tenover F.C. (2003). Pulsed-field gel electrophoresis typing of oxacillin-resistant *Staphylococcus aureus* isolates from the United States: Establishing a national database. J. Clin. Microbiol..

[24] Fey P.D., Endres J.L., Yajjala V.K., Widhelm T.J., Boissy R.J., Bose J.L., Bayles K.W. (2013). A genetic resource for rapid and comprehensive phenotype screening of nonessential *Staphylococcus aureus* genes. mBio.

[25] Horsburgh M.J., Aish J.L., White I.J., Shaw L., Lithgow J.K., Foster S.J. (2002). SigmaB modulates virulence determinant expression and stress resistance: Characterization of a functional *rsbU* strain derived from *Staphylococcus aureus* 8325-4. J. Bacteriol..

[26] Sloan T.J., Murray E., Yokoyama M., Massey R.C., Chan W.C., Bonev B.B., Williams P. (2019). Timing is everything: Impact of naturally occurring *Staphylococcus aureus* AgrC Cytoplasmic Domain Adaptive Mutations on Autoinduction. J. Bacteriol..

[27] Jensen R.O., Winzer K., Clarke S.R., Chan W.C., Williams P. (2008). Differential recognition of *Staphylococcus aureus* quorum-sensing signals depends on both extracellular loops 1 and 2 of the transmembrane sensor AgrC. J. Mol. Biol..

[28] Collins J., Buckling A., Massey R.C. (2008). Identification of factors contributing to T-cell toxicity of *Staphylococcus aureus* clinical isolates. J. Clin. Microbiol..

[29] Laabei M., Recker M., Rudkin J.K., Aldeljawi M., Gulay Z., Sloan T.J., Williams P., Endres J.L., Bayles K.W., Fey P.D. (2014). Predicting the virulence of MRSA from its genome sequence. Genome Res..

[30] Speziale P., Pietrocola G. (2020). The Multivalent Role of Fibronectin-binding Proteins A and B (FnBPA and FnBPB) of *Staphylococcus aureus* in host infections. Front. Microbiol..

[31] Higginson E.E., Galen J.E., Levine M.M., Tennant S.M. (2016). Microgravity as a biological tool to examine host-pathogen interactions and to guide development of therapeutics and preventatives that target pathogenic bacteria. Pathog. Dis..

[32] Hammond T.G., Stodieck L., Birdsall H.H., Becker J.L., Koenig P., Hammond J.S., Gunter M.A., Allen P.L. (2013). Effects of microgravity on the virulence of *Listeria monocytogenes*, *Enterococcus faecalis*, *Candida albicans*, and methicillin-resistant *Staphylococcus aureus*. Astrobiology.

[33] Singh S., Vidyasagar P.B., Kulkarni G.R. (2021). Investigating alterations in the cellular envelope of *Staphylococcus aureus* in simulated microgravity using a random positioning machine. Life Sci. Space Res..

[34] Campbell A.E., McCready-Vangi A.R., Uberoi A., Murga-Garrido S.M., Lovins V.M., White E.K., Pan J.T., Knight S.A.B., Morgenstern A.R., Bianco C. (2023). Variable staphyloxanthin production by *Staphylococcus aureus* drives strain-dependent effects on diabetic wound-healing outcomes. Cell Rep..

[35] Jenul C., Horswill A.R. (2019). Regulation of *Staphylococcus aureus* virulence. Microbiol. Spectr..

[36] Gudeta D.D., Lei M.G., Lee C.Y. (2019). Contribution of *hla* Regulation by SaeR to *Staphylococcus aureus* USA300 Pathogenesis. Infect. Immun..

[37] Crabbe A., Schurr M.J., Monsieurs P., Morici L., Schurr J., Wilson J.W., Ott C.M., Tsaprailis G., Pierson D.L., Stefanyshyn-Piper H. (2011). Transcriptional and proteomic responses of *Pseudomonas aeruginosa* PAO1 to spaceflight conditions involve Hfq regulation and reveal a role for oxygen. Appl. Environ. Microbiol..

[38] Bonnin R.A., Bouloc P. (2015). RNA Degradation in *Staphylococcus aureus*: Diversity of ribonucleases and their impact. Int. J. Genom..

[39] Cheung A.L., Eberhardt K., Heinrichs J.H. (1997). Regulation of protein A synthesis by the *sar* and *agr* loci of *Staphylococcus aureus*. Infect. Immun..

[40] Le K.Y., Otto M. (2015). Quorum-sensing regulation in staphylococci-an overview. Front. Microbiol..

[41] Montgomery C.P., Boyle-Vavra S., Daum R.S. (2010). Importance of the global regulators Agr and SaeRS in the pathogenesis of CA-MRSA USA300 infection. PLoS ONE.

[42] Novick R.P., Geisinger E. (2008). Quorum sensing in staphylococci. Annu. Rev. Genet..

[43] Wang B., Muir T.W. (2016). Regulation of Virulence in *Staphylococcus aureus*: Molecular mechanisms and remaining puzzles. Cell Chem. Biol..

[44] Tan L., Huang Y., Shang W., Yang Y., Peng H., Hu Z., Wang Y., Rao Y., Hu Q., Rao X. (2022). Accessory gene regulator (*agr*) allelic variants in cognate *Staphylococcus aureus* strain display similar phenotypes. Front. Microbiol..

[45] Rudkin J.K., Edwards A.M., Bowden M.G., Brown E.L., Pozzi C., Waters E.M., Chan W.C., Williams P., O’Gara J.P., Massey R.C. (2012). Methicillin resistance reduces the virulence of healthcare-associated methicillin-resistant *Staphylococcus aureus* by interfering with the *agr* quorum sensing system. J. Infect. Dis..

[46] Rudra P., Boyd J.M. (2020). Metabolic control of virulence factor production in *Staphylococcus aureus*. Curr. Opin. Microbiol..

[47] McDowell P., Affas Z., Reynolds C., Holden M.T., Wood S.J., Saint S., Cockayne A., Hill P.J., Dodd C.E., Bycroft B.W. (2001). Structure, activity and evolution of the group I thiolactone peptide quorum-sensing system of *Staphylococcus aureus*. Mol. Microbiol..

[48] Zhao A., Bodine S.P., Xie Q., Wang B., Ram G., Novick R.P., Muir T.W. (2022). Reconstitution of the *S. aureus agr* quorum sensing pathway reveals a direct role for the integral membrane protease MroQ in pheromone biosynthesis. Proc. Natl. Acad. Sci. USA.

[49] Kohn F.P.M., Hauslage J. (2019). The gravity dependence of pharmacodynamics: The integration of lidocaine into membranes in microgravity. npj Microgravity.

[50] Vroom M.M., Rodriguez-Ocasio Y., Lynch J.B., Ruby E.G., Foster J.S. (2021). Modeled microgravity alters lipopolysaccharide and outer membrane vesicle production of the beneficial symbiont *Vibrio fischeri*. npj Microgravity.

[51] Murray E.J., Williams P. (2018). Detection of Agr-Type autoinducing peptides produced by *Staphylococcus aureus*. Methods Mol. Biol..

[52] Junio H.A., Todd D.A., Ettefagh K.A., Ehrmann B.M., Kavanaugh J.S., Horswill A.R., Cech N.B. (2013). Quantitative analysis of autoinducing peptide I (AIP-I) from *Staphylococcus aureus* cultures using ultrahigh performance liquid chromatography-high resolving power mass spectrometry. J. Chromatogr. B Anal. Technol. Biomed. Life Sci..

