# Supplementary material for: Modelled-Microgravity Reduces Virulence Factor Production in Staphylococcus aureus through Downregulation of agr-Dependent Quorum Sensing
Source: Int J Mol Sci. 2023 Nov 6;24(21):15997. doi: 10.3390/ijms242115997 (PMC10648752; doi:10.3390/ijms242115997)
Supplement: Supplementary file 1 [file ijms-24-15997-s001.zip › ijms-2635313-supplementary.pdf]

Modelled-Microgravity Reduces Virulence Factor Production in *Staphylococcus aureus* through Downregulation of *agr*-Dependent Quorum Sensing

Macauley J. Green [1], Ewan J. Murray [2], Paul Williams [2], Amir M. Ghaemmaghami [3], Jonathan W. Aylott [1] and Philip M. Williams [1,*]

[1] School of Pharmacy, University of Nottingham, University Park, Nottingham NG7 2RD, UK; macauley.green@nottingham.ac.uk (M.J.G.)
[2] Biodiscovery Institute and School of Life Sciences, University of Nottingham, University Park, Nottingham NG7 2RD, UK; paul.williams@nottingham.ac.uk (P.W.)
[3] School of Life Sciences, University of Nottingham, University Park, Nottingham NG7 2RD, UK
* Corresponding authors: phil.williams@nottingham.ac.uk (P.M.W.)

Supplementary Material

Fig. S1 Transmission electron microscopy of USA300 (**A**) and SH1000 (**B**)

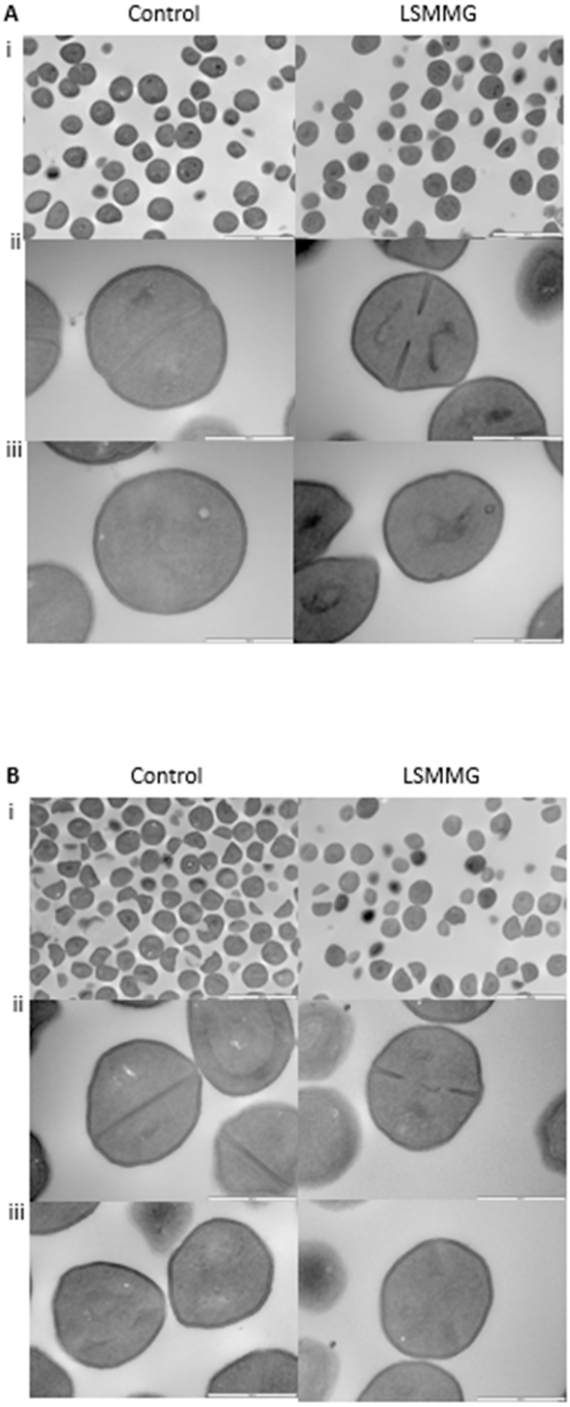

Figure S1: Transmission electron microscopy of USA300 (**A**) and SH1000 (**B**) in the late exponential phase of growth in X-Vivo 15 medium. (i) ×11500, (ii) 60000× of dividing cells and (iii) 60000× magnification of non-dividing cells.
